# Management of a Rare Variant of Hypertrophic Cardiomyopathy

**DOI:** 10.7759/cureus.2074

**Published:** 2018-01-16

**Authors:** Sidra Khalid, Murtaza Sundhu, Alinda Sarma, Bicky Thapa, Praful Maroo

**Affiliations:** 1 Internal Medicine Residency, Fairview Hospital, Cleveland Clinic, USA; 2 Internal Medicine, Fairview Hospital, Cleveland Clinic, USA; 3 Cardiology, Fairview Hospital, Cleveland Clinic, USA

**Keywords:** apical hypertrophic cardiomyopathy, sudden cardiac death, cardiac mri

## Abstract

Apical hypertrophic cardiomyopathy (HCM) is a rare variant of HCM. We present the case of a 26-years-old female who was diagnosed with apical HCM. Her electrocardiogram showed the characteristic T-wave inversions in V2-V5 and her echocardiogram portrayed apical left ventricular hypertrophy. The diagnosis was confirmed with a cardiac magnetic resonance imaging (MRI) scan. She was treated with beta blockers. Our case emphasizes that apical HCM is a relatively benign disease. However, due to the emerging evidence of sudden cardiac deaths in these patients, the risk for sudden death needs to be evaluated.

## Introduction

Hypertrophic cardiomyopathy (HCM) is an autosomal dominant condition that is characterized by segmental or diffuse left ventricular hypertrophy, hyperdynamic left ventricular function, and no evidence of an underlying systemic disease. The left ventricular hypertrophy is manifested as a variety of phenotypes. The rate of occurrence of HCM is about 0.2%, with apical HCM as a rare variant. It is characterized by giant negative T waves on the electrocardiogram. Cardiac magnetic resonance imaging (MRI) is recommended for the confirmation of diagnosis and for risk stratification of sudden cardiac death [1]. Through our case, we aim to highlight the management of patients with apical HCM.

## Case presentation

**Figure 1:**
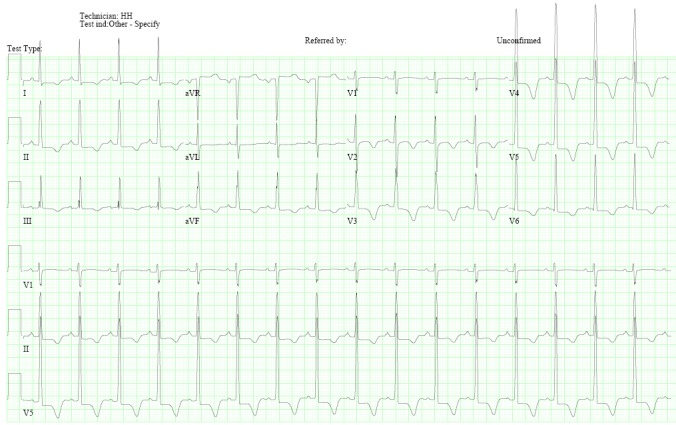
Electrocardiogram: normal sinus rhythm, left ventricular hypertrophy, and T-wave inversions in V2-V5

A 26-year-old female presented with lightheadedness, chest pain, and palpitations for one week. She had a past medical history significant for a cardiac murmur. In her family, her father passed away due to a cardiac event. On examination, her vitals were Temp 98.1 °C, BP 131/84 mmHg, HR 71 /min, RR 15/min, and SpO2 97%. Her physical examination was remarkable for a grade 3/6 systolic murmur at the left lower sternal border, which increased in intensity with standing and Valsalva maneuver. The electrocardiogram showed a normal sinus rhythm with left ventricular hypertrophy and T-wave inversions from V2-V5 (Figure 1).

**Figure 2:**
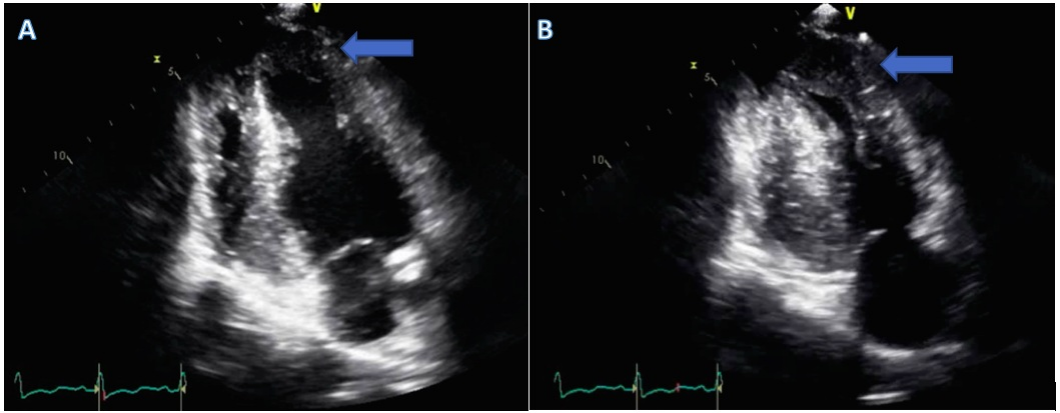
Echocardiogram: apical HCM is seen in diastole (A) and in systole (B) HCM - hypertrophic cardiomyopathy.

**Figure 3:**
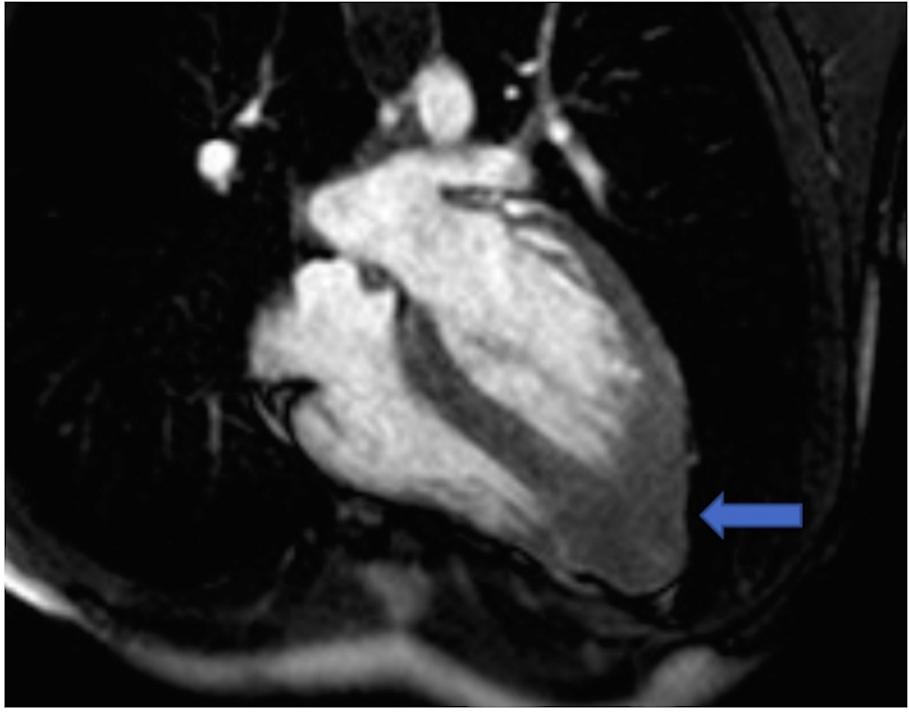
Cardiac MRI: asymmetrical left ventricular hypertrophy characterized by basal, mid inferoseptal, and apical thickness (arrow) MRI - magnetic resonance imaging.

The echocardiogram revealed a left ventricular ejection fraction of 65% with hypertrophy of the apical posterior and lateral walls of the left ventricle suggestive of an apical variant of HCM (Figure 2). Hence, to confirm the diagnosis, a cardiac MRI was performed. Cardiac MRI was consistent with the apical variant of HCM as there was asymmetrical left ventricular hypertrophy, which was delineated by a basal and mid inferoseptal thickness of 1.3 cm and an apical segment of 1.6 cm (Figure 3). There was no evidence of left ventricular outflow obstruction. Moreover, delayed-enhancement imaging revealed subtle mid-myocardial patchy enhancement in the basal, mid inferoseptum, and apical region. Therefore, her management included a beta blocker, with no placement of an implantable cardioverter defibrillator.

## Discussion

Apical HCM has a prevalence of 3% of cases in American cohorts [1]. In apical HCM, the hypertrophy involves the apex of the left ventricle. It most commonly affects middle-aged men [1]. Clinical presentation for patients can vary from being asymptomatic or presenting with chest pain, dyspnea, heart failure, or syncope [2].

Electrocardiogram in an apical HCM patient is characterized by giant negative T waves [1]. Echocardiography is the first line imaging modality to assess for HCM, but it could sometimes lead to false negative results due to poor visualization of the left ventricular apex. Cardiac MRI is, therefore, a useful test to detect apical HCM. The cardiac MRI criteria used to assess apical HCM includes apical wall thickness of more than 15 mm or a ratio of apical to the basal left ventricular wall thickness of 1.3 to 1.5 [1]. Additionally, cardiac MRI could demonstrate a spade-like configuration on the left ventricular long axis images. This is due to the hypertrophied myocardium of the apical anterior and apical posterior walls. In the short axis, the spade-like configuration would be seen as a circumferential hypertrophy of the entire apex [3].

Apical HCM is a relatively benign condition with an overall good prognosis [1]. Most patients do not require long-term specific treatment [3]. Treatment is tailored according to the patient’s symptoms [2]. Due to the emerging evidence of sudden cardiac arrest, fatal arrhythmias, heart failure, and ischemic events in patients with apical HCM, there is a necessity of risk stratifying these patients for sudden cardiac death. We can utilize the risk stratification method used for the patients with HCM. Risk factors would include recurrent unexplained syncope, ventricular tachyarrhythmias, massive hypertrophy (wall thickness of 30 mm or more), previous cardiac arrest, abnormal blood pressure response to a treadmill stress test, high-risk mutant gene, and family history of premature HCM-related sudden death [4]. Patients should undergo assessments for sudden cardiac death annually. An implantable cardioverter defibrillator would be indicated for management when the risk factors are present for sudden cardiac death.

## Conclusions

Apical HCM is a rare form of HCM which has characteristic findings on electrocardiogram, echocardiography, and cardiac MRI. After apical HCM is diagnosed, its management is based on the patient's symptoms and annual risk assessment of sudden cardiac death.
